# Functional impact of gluteal tendinopathy: A secondary cross-sectional analysis of baseline data from the LEAP randomised clinical trial

**DOI:** 10.1016/j.bjpt.2026.101583

**Published:** 2026-03-22

**Authors:** Alison Grimaldi, Anthony Nasser, Rebecca Mellor, Bill Vicenzino

**Affiliations:** a University of Queensland Physiotherapy, St Lucia, Queensland, Australia; b University of Technology Sydney, Graduate School of Health, Australia

**Keywords:** Buttocks, Function, Gluteal tendinopathy, GTPS, Hip, Tendinopathy, Trochanteric bursitis, Outcome measures, *List of Abbreviations:* GT, gluteal tendinopathy, LEAP, LatErAl hip Pain trial, PSFS, patient specific functional scale, RCT, Randomised Clinical Trial, VISA-G (Victorian Institute of Sport Assessment – Gluteal Tendinopathy)

## Abstract

**Background:**

Gluteal tendinopathy (GT) can substantially limit function, although limited information exists on the range of activities affected by GT or the severity of the impact.

**Objective:**

To investigate the type and degree of functional impact in participants with GT.

**Methods:**

A cross-sectional secondary analysis of baseline Patient-Specific Functional Scale (PSFS) data from the multi-centre LatErAl hip Pain (LEAP) randomised clinical trial was performed. Two hundred and four community-dwelling participants (82% female, mean age 55(9) years) were recruited, with GT confirmed through clinical and imaging assessment. Participants nominated and rated three activities most impacted by their condition (0=unable to perform, 10=pre-condition ability). Activities – categorised by type – and degree of impact (mean PSFS score) along with the influence of sex and physical activity were analysed by general linear model. Physical activity levels were derived from the Active Australia Survey.

**Results:**

Data were available for 201 (99%) participants. They nominated 585 PSFS activities over 34 categories. The three most frequently nominated activities were walking (68%), running (27%), and sitting (27%). One hundred and fifty-four (77%) participants were moderately/highly physically active. The mean PSFS score was 4.6(1.9) – with no difference between sexes or physical activity levels (p>0.3). The lowest scores (greatest impact) were for court/field sports (2.5(2.0)), jumping (2.7(2.1)), and martial arts (3.0(2.2)).

**Conclusions:**

GT is characterised by approximately 50% impairment of a range of daily activities (e.g., walking, sitting). PSFS activities nominated by patients should be considered in treatment and measuring outcome.

## Highlights

Participants with gluteal tendinopathy report:

• Walking, running, sitting and sleeping as being most affected.
• Being less able to perform field sports, jumping, and martial arts.
• Experiencing substantial impact of their condition on common activities.

## Introduction

Gluteal tendinopathy (GT) is a common cause of lateral hip pain that can substantially limit function, particularly in peri- and post-menopausal women.^1^^,^^2^ Sleep, single-leg standing, walking, stair climbing, and rising from sitting are commonly affected and are typically included in GT-specific patient-rated outcome measures, such as the Victorian Institute of Sport Assessment – Gluteal Tendinopathy (VISA-G)^3^ and Lateral Hip Pain Questionnaires.^4^

Limited information exists on the range of activities affected by GT or the severity of the impact. Existing GT-specific outcome measures may not fully capture the breadth of functional limitations experienced by individuals. The Patient Specific Functional Scale (PSFS) allows participants to nominate personally meaningful activities affected by their condition and rate the impairment severity.^5^ A previous mediation analysis from the LatErAl hip Pain (LEAP) randomised clinical trial (RCT), revealed that change in PSFS scores significantly mediated differences in treatment effects.^6^ Greater focus on improving patient-specific functional limitations may contribute to more successful and meaningful outcomes for the individual.

This study explored the type of participant nominated PSFS activities, their frequency, and degree to which they were affected. We hypothesised that GT would be characterised by a range of nominated PSFS activities and PSFS scores – influenced by participant physical activity level not sex.

## Methods

### Design

This was a secondary cross-sectional analysis of baseline data from the LEAP RCT, a prospective three-arm trial (n= 204) in Australia. The LEAP trial protocol was prospectively published,^4^ registered (ACTRN12612001126808) and approved by the University of Queensland (#2012000930) and University of Melbourne (ID 1238598) ethics committees. All participants provided written informed consent. Trial findings have been reported elsewhere.^7^ The study was reported in accordance with the Strobe (Strengthening the Reporting of Observational Studies in Epidemiology) guidelines.^8^

### Participants

Adults aged 35-70 years with lateral hip pain ≥4/10 for > 3 months, were recruited from the community via advertisements in print, radio, and social media. GT diagnosis was confirmed clinically and with imaging. Full inclusion/exclusion criteria are in the protocol.^4^ All participants with available baseline PSFS data were included.

### Baseline data

a). Participant characteristics

At baseline, participants completed demographic and condition-related surveys, including pain severity and duration. Pain severity (previous week) was measured using a numeric rating scale (0 = no pain, 10 = worst pain imaginable). Pain duration was recorded in months.

b). Participant physical activity levels

Participant physical activity levels were also collected at baseline using the Active Australia Survey.^9^ The Active Australia Survey measures the number of minutes spent walking, and undertaking moderate activity, vigorous activity and vigorous gardening or yard work, over the previous week. Total minutes of activity can be multiplied by an intensity factor (walking x 3.5; moderate activity x 5; vigorous activity x 7.5) and summed for a total score. This allows categorisation of activity levels as sedentary (<50), low (50-799), moderate (800-1600+ but with < 1 h of vigorous activity) and high (>1,600 with 1 h or more of vigorous physical activity) according to the Australian Bureau of Statistics.^10^

c). PSFS

The PSFS measures how a health condition affects a person’s ability to do things/activities.^5^ Participants responded to the stimulus: *‘I am going to ask you about 3 activities that are important to you, that you are unable to do, or are having difficulty with. Can you rate your ability to do (each of) these activities between 0 and 10, where 0 is ‘unable to perform the activity’, and 10 is ‘able to perform at the same level as before the injury or problem’.* If participants could not nominate an activity, assessors encouraged them; if still unable, the field was left blank. The mean of the scores is the PSFS score.

### Data analysis

Two independent researchers independently grouped PSFS activities into categories (e.g., running/jogging, see Appendix A). Categories were not pre-defined but were developed inductively. Discrepancies were resolved by discussion to minimise classification bias. Activities were summarised by type, frequency, and mean PSFS score. Continuous variables were reported as means (± standard deviation (SD), 95% confidence intervals (CI)), categorical variables as counts and percentages – and where appropriate, plotted for visual representation/inspection. Distribution of PSFS score data was assessed by visualisation of QQ plot and the Shapiro-Wilk test. The effect of sex and physical activity levels on PSFS score was analysed with general linear models in SPSS software (IBM SPSS Statistics Version 29.0.1.0 (171)).

## Results

### Participants

Baseline PSFS data was available for 201/204 (99%) participants (82% female, mean age 55(9) years), mean pain severity was 4.9(1.0) and median duration was 24 months (IQR 8–48, range 3–192). In the week prior to baseline measures, participants reported completing an average of 463 min of physical activity, with 234 of those minutes spent walking and 168 min in some form of vigorous activity. Numbers (percentage) of participants falling into each activity level category were as follows: level 1-sedentary, 4 (2%); level 2-low, 43 (21%); level 3-moderate, 66 (33%); and level 4-high, 88 (44%). Males had a slightly higher Active Australia Survey score (3.5(0.7)) than females (3.1(0.9)) – mean difference 0.4 (CI: 0.1, 0.7, p=0.02).

### PSFS responses

Three PSFS activities were nominated by 184 (90%) participants, two by ten (5%) participants, and one by seven (3%) participants. Three (1%) participants did not nominate any activities. Six participants listed two activities in one response; these were counted separately.

### Type and frequency of participant nominated PSFS activities

Of the 585 PSFS activities reported, 383 (65%) were related to physical activities (e.g., walking, running, gardening) and 202 (35%) were activities-of-daily-living (e.g., sitting, side lying, sleeping, stairs).

**Fig. 1:**
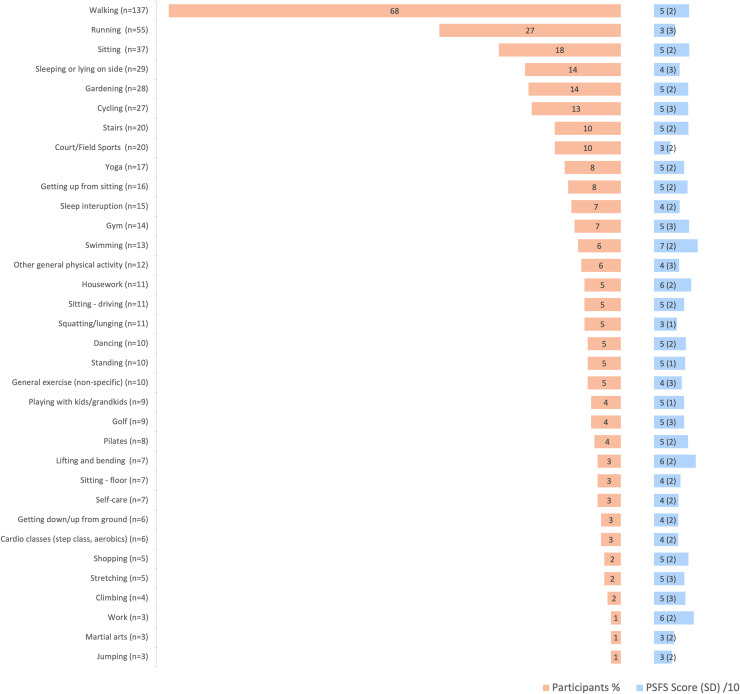
Frequency (%) of participant nominated Patient Specific Functional Scale (PSFS) activities and their PSFS (SD) scores, where 0 is ‘unable to perform the activity’, and 10 is ‘able to perform at the same level as before the injury or problem’.

Activities were divided into 34 categories (Fig. 1). The most common activity impacted by GT was walking, with 137 (68%) participants mentioning walking, followed by running (55 (27%) responses), sitting (54 (27%)) and sleeping (44 (22%)). Sitting responses included ‘sitting’ (37/201; 18%), ‘sitting driving’ (11/201; 5%) and ‘sitting on the floor’ (7/201; 3%). Sleeping responses included sleeping or lying on the side (29/201; 14%), and sleep interruption (15/201; 7%). Court and field sports included tennis, hockey, football, netball, soccer, badminton, table tennis, and lawn bowls. Activities mentioned ≤ three times (horse riding, skiing, sex, bodyboarding, rowing, camping, fishing, water skiing, and volunteering) were grouped as ‘other general physical activity’.

### Ability to perform nominated PSFS activities (PSFS Scores)

The PSFS score was normally distributed (Shapiro-Wilk test: p=0.2) with a mean of 4.6/10 (1.9) (CI: 4.4, 4.9)). The mean PSFS scores for the five most frequently nominated activities were: walking 5.3(2.2), running 3.2(2.7), sitting 5.0(2.0), sleeping on side 3.9(2.5) and gardening 5.2(2.7) (Fig. 1). The activity with the lowest mean PSFS score (lowest ability/greatest impact) was court/field sports (2.5(2.0)), followed by jumping (2.7(2.1)), martial arts (3.0(2.2)), and running (3.2(2.7)). The activities with the highest mean scores were swimming (6.6(2.1)), lifting/bending (6.3(2.1)), and work (6.0(2.2)).

### Influence of sex and activity levels on PSFS scores

There was no difference between the female and male participants in their mean PSFS score (4.6(1.9) versus 4.8(2.1) respectively, p=0.52). The mean PSFS score between the four activity levels with sex as a covariate was not different (p=0.31) with sedentary scoring 4.1 (CI: 2.0, 6.1, n=4), low 4.3 (CI: 3.7,4.9, n=43), moderate 4.5 (CI: 4.0,4.9, n=66) and high 4.9 (CI: 4.5,5.3, n=88).

### Comparison of PSFS activities across active Australia survey activity levels

**Fig. 2:**
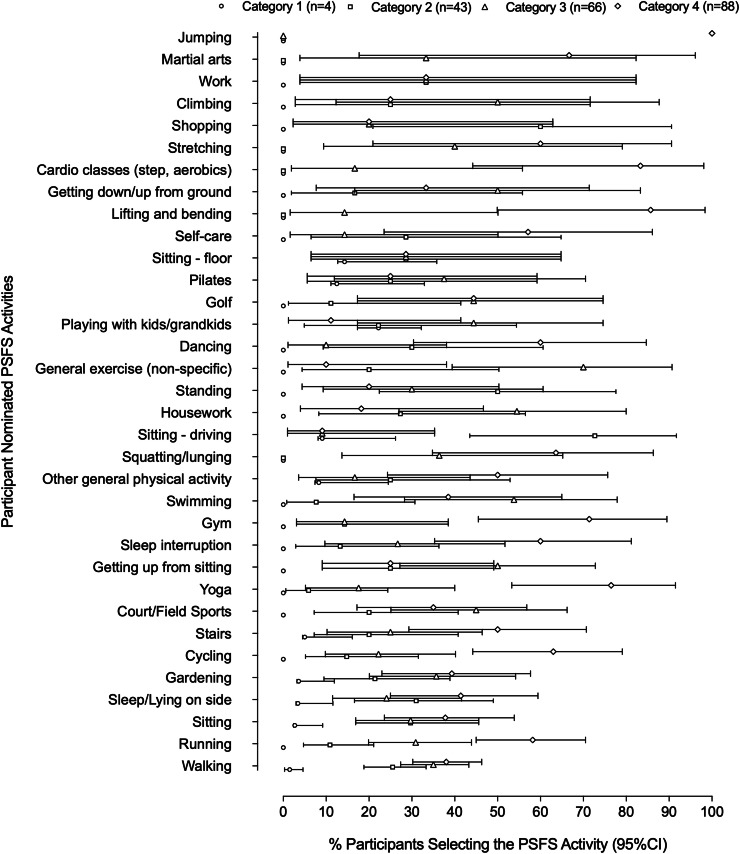
Percentage (95% CI error bars) of participants nominating PSFS activities per category of the Active Australia Survey, where Category 1 through 4 are deemed to be sedentary, low, moderate and high levels of physical activity. Note 0 and 100% have 0 as their 95%CI.

Fig. 2 shows that there is much overlap in data (mean, confidence intervals) for the three higher physical activity levels with a trend to having those in the higher categories nominating higher load activities, such as, cardio, running, gym, jumping, lifting, and bending. In contrast, Category 1 participants - though fewer in number - showed a trend to nominate lower load activities, for example, sitting on floor, gardening, walking, stairs, driving, and playing with grandchildren.

## Discussion

Walking, running, sitting and sleeping were the most reported functional tasks impacted by GT. The functional limitation imposed by GT was greater for more vigorous weightbearing activities (field sports, running) compared with less vigorous activities (shopping, walking, swimming). This is the first study to report on specific functional limitations experienced by those with GT. The relatively high frequency and degree of impact of GT on vigorous activities may be under-recognised and inadequately addressed in clinical practice.

No other GT RCTs have reported collecting PSFS data. One GT study used the PSFS as part of their translation and cross-cultural validation study for the VISA-G.^11^ They reported a mean score of 4.3 – 4.62, similar to that of our LEAP cohort, but did not provide any descriptive data around types of activities. We found that the PSFS score was not influenced by the participants’ physical activity levels when accounting for a small difference in favour of higher physical activity levels in males. This suggests that the PSFS score has clinical utility across different levels of regular physical activity of those with GT.

Two RCTs studying other lower limb tendinopathies have used the PSFS to collect functional data in their populations. A study comparing exercise interventions for overweight females, aged 40-50 years, with Achilles tendinopathy reported an average PSFS score of 5.07 – 5.25, a little higher than the LEAP cohort at 4.6/10.^12^ One other RCT of younger males with patellar tendinopathy reported an average PSFS score of 6.2 – 6.3.^13^ Neither study reported the specifics of functional tasks that participants nominated within the PSFS. The PSFS is an underutilised tool in lower limb tendinopathy research. Further implementation and descriptive reporting of PSFS results may provide further clarity around the types of meaningful tasks that are impacted by tendinopathy, with relevance to development of optimally targeted goals and individualised interventions.

Only one other pilot RCT collected physical activity data from participants with GT, using The International Physical Activity Questionnaire Short Form.^14^ The study reported no significant change in activity level across the 3-month intervention but did not report data regarding levels of activity. There is inadequate information available regarding activity levels in GT populations. Despite this group often being characterised as ‘sedentary’, 77% of LEAP trial participants were moderately – highly active, including on average almost three hours of vigorous activity per week. High activity levels, particularly rapid increases in activity levels, may contribute to the onset of symptoms related to GT. In a clinical setting, individual activity levels should be measured and appropriate load management education provided. Furthermore, those with GT may have higher level activity goals than assumed, which may require more advanced rehabilitation to facilitate such goals.

With regard to specific functional limitations, considering walking and running were two of the most frequently impacted activities, clinicians should ensure they include specific load-modification education and gait training when relevant. Physiotherapists may not currently focus enough attention on addressing such specific functional deficits. One survey of physiotherapists in the United Kingdom reported that only 51.4% of respondents always-or-often provided gait training and only 62% always-or-often provided functional movement training.^15^ The LEAP mediation analysis that revealed that change in functional ability (PSFS scores) was a significant mediator of differences in treatment effects^6^ suggests that patient-centred treatment should include assessment and rehabilitation of self-nominated tasks and targeted programs to return to those activities.

### Study limitations

A strength of the study was the blinded PSFS data collection from the largest GT RCT to date. Item categorisation was somewhat subjective, although items were not overly collapsed (34 categories), to avoid losing meaningful information. The uncategorised data can be found in Appendix B. The predominance of female participants in our study reflects the typical sex distribution of GT but may limit the generalisability of findings to males. Additionally, the cross-sectional design limits causal inference, and findings may not generalise beyond similar clinical populations.

## Conclusion

The baseline PSFS data of 201 participants of the LEAP RCT has highlighted that GT substantially impacts patients’ ability to perform a wide range of functional activities, from activities-of-daily-living to sporting activities. As a large variety of different activities were nominated by participants, it underscores the need for personalised treatment plans.

## Declaration of competing interest

The authors declare no competing interest.

## Appendix Supplementary materials

Image, application 1
