# Supplementary material for: Functional impact of gluteal tendinopathy: A secondary cross-sectional analysis of baseline data from the LEAP randomised clinical trial
Source: Braz J Phys Ther. 2026 Mar 22;30(3):101583. doi: 10.1016/j.bjpt.2026.101583 (PMC13227185; doi:10.1016/j.bjpt.2026.101583)
Supplement: Supplementary file 1 [file mmc1.docx]

**Appendix A:** Patient Specific Functional Scale

| **Category** | **Mean PSFS** | **Frequency category mentioned** | **Standard deviation** | **Range of PSFS scores** | **Median PSFS score** |
| --- | --- | --- | --- | --- | --- |
| Jumping | 2.7 | 3 | 2.1 | 0-6 | 3 |
| Martial arts | 3 | 3 | 2.16 | 0-5 | 4 |
| Work | 6 | 3 | 2.16 | 0-5 | 5 |
| Climbing | 4.75 | 4 | 2.95 | 0-9 | 5.5 |
| Stretching | 4.6 | 5 | 3.2 | 1-6 | 4 |
| Shopping | 5.2 | 5 | 1.6 | 0-10 | 5 |
| Exercise classes (e.g. step class, aerobics) | 3.667 | 6 | 2.49 | 0-7 | 3.5 |
| Getting down/up from ground | 3.667 | 6 | 2.21 | 1-8 | 3.5 |
| Self-care | 3.714 | 7 | 2.19 | 2-7 | 4 |
| Sitting - floor | 4 | 7 | 1.69 | 0-9 | 3 |
| Lifting and bending | 6.3 | 7 | 2.12 | 0-6 | 6 |
| Exercise class - Pilates | 5.125 | 8 | 2.15 | 2-7 | 5 |
| Golf | 4.556 | 9 | 3.02 | 0-10 | 5 |
| Playing with kids/grandkids | 4.556 | 9 | 1.26 | 2-8 | 4 |
| General exercise (non-specific) | 4.2 | 10 | 2.71 | 0-8 | 4 |
| Standing | 4.7 | 10 | 1.42 | 0-10 | 5 |
| Dancing | 4.8 | 10 | 2.18 | 3-7 | 5 |
| squatting/lunging | 3.455 | 11 | 1.37 | 0-10 | 4 |
| Sitting - driving | 4.545 | 11 | 1.97 | 2-8 | 4 |
| Housework | 5.636 | 11 | 2.27 | 0-8 | 6 |
| Other general physical activity | 3.8 | 12 | 2.7 | 0-9 | 4.5 |
| Swimming | 6.615 | 13 | 2.1 | 2-8 | 6 |
| Gym | 5.286 | 14 | 2.79 | 1-8 | 5 |
| Sleep interruption | 3.867 | 15 | 1.71 | 0-9 | 4 |
| Getting up from sitting | 5.063 | 16 | 1.89 | 0-9 | 5.5 |
| Exercise class - yoga | 4.545 | 17 | 2.42 | 3-8 | 5 |
| Court/Field Sports (tennis, hockey, football, netball, badminton, table tennis) | 2.5 | 20 | 2.04 | 2-8 | 2 |
| Stairs | 5.2 | 20 | 1.83 | 0-10 | 5 |
| Cycling | 5.185 | 27 | 2.72 | 0-10 | 6 |
| Gardening | 5.179 | 28 | 2.32 | 1-9 | 5 |
| Sleeping/Lying on side | 3.862 | 29 | 2.52 | 1-9 | 3 |
| Sitting - general | 5.324 | 37 | 1.93 | 4-9 | 5 |
| Running (includes jogging) | 3.2 | 55 | 2.71 | 3-8 | 3 |
| Walking | 5.299 | 137 | 2.15 | 2-10 | 5 |
| PSFS = Patient specific functional scale | | | | | |

**Appendix B**

| **Construct Group** | **Activity** | **PSFS Score** |
| --- | --- | --- |
| Climbing | Climbing out of a swimming pool | 0 |
| Climbing | Climb ladder | 8 |
| Climbing | Going up/down ladder | 6 |
| Climbing | Climbing | 5 |
| Court/Field Sports | Tennis | 0 |
| Court/Field Sports | Tennis | 5 |
| Court/Field Sports | Playing / training for football | 2 |
| Court/Field Sports | Playing hockey | 0 |
| Court/Field Sports | Tennis | 0 |
| Court/Field Sports | Playing competitive tennis | 3 |
| Court/Field Sports | Netball | 0 |
| Court/Field Sports | Playing tennis | 0 |
| Court/Field Sports | Tennis | 5 |
| Court/Field Sports | Hockey | 3 |
| Court/Field Sports | Bowls | 5 |
| Court/Field Sports | Regular tennis | 2 |
| Court/Field Sports | Badminton | 5 |
| Court/Field Sports | Playing table tennis | 2 |
| Court/Field Sports | Lawn bowls | 2 |
| Court/Field Sports | Soccer with son | 6 |
| Court/Field Sports | Jogging - tennis | 5 |
| Court/Field Sports | Stiffness when playing tennis | 3 |
| Court/Field Sports | Soccer/sprinting as part of a sport | 2 |
| Court/Field Sports | Tennis | 0 |
| Cycling | Bicycle riding | 1 |
| Cycling | Cycling | 9 |
| Cycling | Cycling | 7 |
| Cycling | Bicycling | 1 |
| Cycling | Bike riding | 9 |
| Cycling | Bike riding | 6 |
| Cycling | Cycling on exercise bike | 5 |
| Cycling | Cycling up hills | 7 |
| Cycling | Cycling | 9 |
| Cycling | Bicycle riding | 4 |
| Cycling | Cycling | 7 |
| Cycling | Cycling | 4 |
| Cycling | Ride bike | 3 |
| Cycling | Riding bike | 3 |
| Cycling | Riding a bike | 7 |
| Cycling | Cycling | 7 |
| Cycling | Bike riding | 7 |
| Cycling | Bike riding | 9 |
| Cycling | Cycling | 5 |
| Cycling | Cycling | 6 |
| Cycling | Sprint cycling | 6 |
| Cycling | Cycle | 2 |
| Cycling | Cycling | 6 |
| Cycling | Riding | 0 |
| Cycling | Cycling | 0 |
| Cycling | Spin class | 7 |
| Cycling | Cycling | 3 |
| Dancing | Ballroom dancing | 5 |
| Dancing | Dancing | 5 |
| Dancing | Dancing | 3 |
| Dancing | Teaching ballet | 6 |
| Dancing | Dancing | 5 |
| Dancing | Dancing | 6 |
| Dancing | Zumba dance class | 0 |
| Dancing | Line dancing | 9 |
| Dancing | Dancing | 4 |
| Dancing | Dance | 5 |
| Exercise classes | Step aerobics | 5 |
| Exercise classes | Step Aerobics/Body pump | 2 |
| Exercise classes | Classes at gym | 7 |
| Exercise classes | High impact aerobics | 0 |
| Exercise classes | Aerobics | 6 |
| Exercise classes | Gym classes 3 times a week | 2 |
| Exercise classes - Pilates | Pilates | 5 |
| Exercise classes - Pilates | Pilates | 7 |
| Exercise classes - Pilates | Pilates | 5 |
| Exercise classes - Pilates | Pilates class | 1 |
| Exercise classes - Pilates | Pilates | 8 |
| Exercise classes - Pilates | Pilates | 5 |
| Exercise classes - Pilates | Pilates | 3 |
| Exercise classes - Pilates | Pilates | 7 |
| Exercise classes - Yoga | Yoga | 2 |
| Exercise classes - Yoga | Yoga - certain poses | 3 |
| Exercise classes - Yoga | Yoga | 1 |
| Exercise classes - Yoga | Yoga | 3 |
| Exercise classes - Yoga | Yoga | 8 |
| Exercise classes - Yoga | Yoga - some exercises more difficult | 5 |
| Exercise classes - Yoga | Yoga | 7 |
| Exercise classes - Yoga | Yoga | 0 |
| Exercise classes - Yoga | Yoga | 6 |
| Exercise classes - Yoga | Yoga | 5 |
| Exercise classes - Yoga | Yoga | 2 |
| Exercise classes - Yoga | Yoga | 7 |
| Exercise classes - Yoga | Yoga | 2 |
| Exercise classes - Yoga | Yoga | 7 |
| Exercise classes - Yoga | Yoga | 3 |
| Exercise classes - Yoga | Yoga | 7 |
| Exercise classes - Yoga | Yoga | 6 |
| Gardening | Gardening | 9 |
| Gardening | Mowing lawns | 0 |
| Gardening | Gardening | 5 |
| Gardening | Gardening | 4 |
| Gardening | Heavy garden work on uneven ground | 1 |
| Gardening | Gardening | 0 |
| Gardening | Gardening/heavy yard work for several hrs a day | 5 |
| Gardening | Gardening | 7 |
| Gardening | Gardening | 3 |
| Gardening | Gardening | 2 |
| Gardening | Gardening for long periods, involving squatting and getting up and down | 6 |
| Gardening | Gardening | 6 |
| Gardening | Bending over gardening | 5 |
| Gardening | Manual labour around house/yard | 4 |
| Gardening | Gardening | 5 |
| Gardening | Gardening | 7 |
| Gardening | Gardening | 5 |
| Gardening | Gardening | 5 |
| Gardening | Gardening | 9 |
| Gardening | Gardening | 8 |
| Gardening | Gardening | 7 |
| Gardening | Gardening | 5 |
| Gardening | Gardening | 8 |
| Gardening | Gardening | 5 |
| Gardening | Gardening | 6 |
| Gardening | Mowing the grass | 6 |
| Gardening | Gardening | 7 |
| Gardening | Gardening | 5 |
| General Exercise | Participate in regular exercise, walking (no gym) | 3 |
| General exercise | Aerobic exercise | 5 |
| General exercise | Vigorous exercise | 5 |
| General exercise | Exercise | 10 |
| General exercise | Exercise hard | 2 |
| General Exercise | Exercise | 2 |
| General exercise | Exercise (gentle) | 7 |
| General exercise | Exercise | 5 |
| General exercise | Exercise | 3 |
| General exercise | Aerobic exercise | 0 |
| Getting down/up from ground | Sitting on floor cross legged then getting up | 4 |
| Getting down/up from ground | Getting down and up again from gardening | 2 |
| Getting down/up from ground | Kneeling, rising | 4 |
| Getting down/up from ground | Getting up or down from lying/kneeling | 1 |
| Getting down/up from ground | Crouch down and get up easily | 8 |
| Getting down/up from ground | Getting up from floor | 3 |
| Getting up from sitting | Getting out of car after long journey | 5 |
| Getting up from sitting | Standing up from sitting | 8 |
| Getting up from sitting | Getting up after sitting | 6 |
| Getting up from sitting | Sit for long periods, then get up and walk | 2 |
| Getting up from sitting | Walking after sitting | 8 |
| Getting up from sitting | Getting up after sitting for 30 minutes | 4 |
| Getting up from sitting | Getting into car after walking | 7 |
| Getting up from sitting | Standing from the car or chair after 15 minutes | 6 |
| Getting up from sitting | Getting up from sitting without limping | 4 |
| Getting up from sitting | Standing from sitting | 6 |
| Getting up from sitting | If sitting for >30 mins, stiffness getting up | 2 |
| Getting up from sitting | Standing from sitting | 6 |
| Getting up from sitting | Getting out of bed | 2 |
| Getting up from sitting | Standing up and walking after sitting for 15-20 minutes | 5 |
| Getting up from sitting | Getting up from seated position | 4 |
| Getting up from sitting | Getting up from a seated position | 6 |
| Golf | Golf | 5 |
| Golf | Play golf | 7 |
| Golf | Golf | 0 |
| Golf | Golf | 5 |
| Golf | Golf | 10 |
| Golf | Golf | 7 |
| Golf | Golf | 2 |
| Golf | Golf | 4 |
| Golf | Golf | 1 |
| Gym | Exercise, Gym, Weights | 5 |
| Gym | Gym | 1 |
| Gym | Weightlifting | 0 |
| Gym | Weight training | 10 |
| Gym | Gym exercise plan | 2 |
| Gym | Gym | 7 |
| Gym | Normal gym exercises | 6 |
| Gym | Being active at gym | 5 |
| Gym | Gym | 6 |
| Gym | Cross Trainer | 5 |
| Gym | Gym | 5 |
| Gym | Weight training | 5 |
| Gym | Gym | 10 |
| Gym | Weights | 7 |
| Other general physical activity | Horse riding | 0 |
| Other general physical activity | Horse riding | 4 |
| Housework | Sweeping/mopping house | 4 |
| Housework | Vacuuming | 5 |
| Housework | Housework | 9 |
| Housework | Vacuuming | 1 |
| Housework | Housework | 6 |
| Housework | Housework/vacuuming | 6 |
| Housework | House chores | 7 |
| Housework | Housework | 5 |
| Housework | House chores | 8 |
| Housework | Housework e.g. vacuuming, bedmaking | 3 |
| Housework | Housework | 8 |
| Lifting and bending | Bending to floor | 3 |
| Lifting and bending | Crouching down | 8 |
| Lifting and bending | Lifting | 6 |
| Lifting and bending | Lifting | 8 |
| Lifting and bending | Lifting and bending | 3 |
| Lifting and bending | Heavy manual lifting | 3 |
| Lifting and bending | Bending | 6 |
| Martial arts | Karate side kicks | 5 |
| Martial arts | Karate | 4 |
| Martial Arts | Martial Arts - Aikido falls | 0 |
| Other general physical activity | Exercise eg X-C skiing | 0 |
| Other general physical activity | Snow skiing | 5 |
| Jumping | Jumping | 5 |
| Jumping | Jumping | 0 |
| Jumping | Jumping | 3 |
| Other general physical activity | Bodyboarding | 3 |
| Other general physical activity | Fishing in a small boat | 4 |
| Other general physical activity | Water skiing | 10 |
| Other general physical activity | Rowing | 7 |
| Other general physical activity | Camping | 0 |
| Other general physical activity | Volunteering | 3 |
| Other general physical activity | Loving | 4 |
| Other general physical activity | Sex | 5 |
| Playing with kids/grandkids | Playing with the kids | 4 |
| Playing with kids/grandkids | Being able to play on floor with grandchild and get up again | 3 |
| playing with kids/grandkids | Playing with kids at park | 6 |
| playing with kids/grandkids | Playing with kids | 4 |
| Playing with kids/grandkids | Jumping up quickly with kids | 4 |
| Playing with kids/grandkids | Activity with playing with grandchildren | 3 |
| playing with kids/grandkids | Playing actively with my 5 yr old | 7 |
| playing with kids/grandkids | Activities with my children | 5 |
| Playing with kids/grandkids | Playing with kids | 5 |
| Running | jogging | 8 |
| Running | Jogging | 8 |
| Running | Jogging | 5 |
| Running | Jogging | 8 |
| Running | Jogging | 5 |
| Running | Jogging | 5 |
| Running | Jogging | 0 |
| Running | Long distance running | 7 |
| Running | Long distance running | 7 |
| Running | Long distant running | 0 |
| Running | Run | 3 |
| Running | Run in an emergency at work | 2 |
| Running | Running | 7 |
| Running | Running | 6 |
| Running | Running | 6 |
| Running | Running | 4 |
| Running | Running | 0 |
| Running | Running | 0 |
| Running | Running | 3 |
| Running | Running | 2 |
| Running | Running | 6 |
| Running | Running | 4 |
| Running | Running | 8 |
| Running | Running | 0 |
| Running | Running | 0 |
| Running | running | 1 |
| Running | Running | 2 |
| Running | Running | 1 |
| Running | Running | 0 |
| Running | Running | 2 |
| Running | Running | 9 |
| Running | Running | 6 |
| Running | Running | 1 |
| Running | Running | 1 |
| Running | Running | 3 |
| Running | Running | 4 |
| Running | Running | 2 |
| Running | Running | 5 |
| Running | Running | 4 |
| Running | Running | 0 |
| Running | Running | 3 |
| Running | Running | 4 |
| Running | Running | 0 |
| Running | Running | 4 |
| Running | Running | 0 |
| Running | Running | 1 |
| Running | Running | 2 |
| Running | Running | 0 |
| Running | Running | 0 |
| Running | Running | 2 |
| Running | Running | 0 |
| Running | Running | 1 |
| Running | Running | 3 |
| Running | Running up and down hills | 4 |
| Running | Sprinting | 7 |
| Self-care | Putting underwear and trousers | 7 |
| Self-care | Putting on pantyhose on long pants | 2 |
| Self-care | Put shoes and socks on without struggle | 4 |
| Self-care | Holding leg out straight while standing on other leg | 4 |
| Self-care | Putting on pants | 6 |
| Self-care | Dressing - bending to put on tights or doing up zippers | 3 |
| Self-care | Taking a bath | 0 |
| Shopping | Shopping | 3 |
| Shopping | Shopping for more than 1.5 hours | 5 |
| Shopping | Shopping (mainly difficulty is twisting to push trolley) | 8 |
| Shopping | Shopping | 5 |
| Shopping | Shopping | 5 |
| Sitting | Sitting - computer, reading | 7 |
| Sitting | Sitting in a car or chair for long periods | 7 |
| Sitting | Sitting at my desk for long periods | 4 |
| Sitting | Sitting and working at my microscope | 5 |
| Sitting | Sitting for long periods | 7 |
| Sitting | working - sitting long periods | 8 |
| Sitting | Sitting | 5 |
| Sitting | Sitting for 30 mins | 7 |
| Sitting | Sitting reading or studying | 6 |
| Sitting | Sitting in the car | 7 |
| Sitting | Relaxing in lounge, watching TV without pain | 2 |
| Sitting | Sitting - reading, watching TV | 5 |
| Sitting | Getting in/out of car | 5 |
| Sitting | Sitting in chair | 7 |
| Sitting | Sitting to complete class planning | 6 |
| Sitting | Sitting | 5 |
| Sitting | Sitting for long periods | 4 |
| Sitting | Sitting for an hour | 5 |
| Sitting | Sitting on floor for half hour | 3 |
| Sitting | Sewing at machine | 7 |
| Sitting | Sitting comfortably through a movie | 4 |
| Sitting | Using computer | 7 |
| Sitting | Sitting at a desk | 5 |
| Sitting | Sit comfortably without pain | 2 |
| Sitting | Sitting for 30 mins | 2 |
| Sitting | Sitting long periods in study | 6 |
| Sitting | Sitting in uncomfortable chair | 9 |
| Sitting | Sitting still e.g. movies | 1 |
| Sitting | Sitting for too long | 7 |
| Sitting | Sitting for long periods | 3 |
| Sitting | Sitting in car > 1 hr | 5 |
| Sitting | Sit with ankle resting on knee | 5 |
| Sitting | Sitting for extended periods | 4 |
| Sitting | Sitting | 8 |
| Sitting | Sitting watching a movie | 6 |
| Sitting | Sitting with crossed legs | 8 |
| Sitting | Watching a movie at the cinema | 3 |
| Sitting - Driving | Driving for work | 4 |
| Sitting - Driving | Driving distances | 8 |
| Sitting - Driving | Driving car some distance without pain | 2 |
| Sitting - Driving | Driving long distance | 5 |
| Sitting - Driving | Driving long distances | 4 |
| Sitting - Driving | Driving | 5 |
| Sitting - Driving | Long distance driving | 3 |
| Sitting - Driving | Angle of hip in car seat | 7 |
| Sitting - Driving | Driving in car for more than 30 mins | 7 |
| Sitting - Driving | Driving | 3 |
| Sitting - Driving | Driving | 2 |
| Sitting - floor | Sitting on ground | 2 |
| Sitting - floor | Sitting on the floor with legs crossed | 6 |
| Sitting - floor | Meditating | 4 |
| Sitting - floor | Cross legged sitting on calves | 7 |
| Sitting - floor | Sitting on floor | 3 |
| Sitting - floor | Sitting on wooden floor | 3 |
| Sitting - floor | Sit cross legged with grandchildren | 3 |
| Sleep Interruption | Sleeping uninterrupted | 5 |
| Sleep Interruption | Wakes me up sometimes | 5 |
| Sleep Interruption | Sleep uninterrupted by hip pain | 5 |
| Sleep Interruption | Sleep for more than 2 hours | 1 |
| Sleep Interruption | Getting to sleep | 3 |
| Sleep Interruption | Sleeping uninterrupted | 4 |
| Sleep Interruption | Sleeping | 5 |
| Sleep Interruption | Sleeping through the night | 0 |
| Sleep Interruption | Sleeping | 6 |
| Sleep Interruption | Sleeping | 5 |
| Sleep Interruption | Sleeping uninterrupted | 4 |
| Sleep Interruption | sleeping | 6 |
| Sleep Interruption | Sleeping undisturbed | 3 |
| Sleep Interruption | Sleeping | 4 |
| Sleep Interruption | Sleeping | 2 |
| Sleeping/Lying on side | Sleep on right side | 1 |
| Sleeping/Lying on side | Sleeping | 5 |
| Sleeping/Lying on side | Sleeping on side with sore hip exposed | 5 |
| Sleeping/Lying on side | Sleeping on left side | 0 |
| Sleeping/Lying on side | Lying on the left side (especially in the morning) | 2 |
| Sleeping/Lying on side | Lying on my left side in bed | 2 |
| Sleeping/Lying on side | Sleeping on my side | 7 |
| Sleeping/Lying on side | Lying on side in bed | 5 |
| Sleeping/Lying on side | Lye on side | 8 |
| Sleeping/Lying on side | Lying on sleep | 1 |
| Sleeping/Lying on side | Sleeping on side for >2 hrs | 2 |
| Sleeping/Lying on side | Lying on left side | 0 |
| Sleeping/Lying on side | Lying on my side | 2 |
| Sleeping/Lying on side | Lying on side | 8 |
| Sleeping/Lying on side | Raise my leg while hying on my left side | 3 |
| Sleeping/Lying on side | Lying on Side | 6 |
| Sleeping/Lying on side | Lie on sides | 3 |
| Sleeping/Lying on side | Lying on right | 1 |
| Sleeping/Lying on side | Lying on left side overnight | 5 |
| Sleeping/Lying on side | Sleeping on my side | 9 |
| Sleeping/Lying on side | Lying on side | 3 |
| Sleeping/Lying on side | Lying on my side | 6 |
| Sleeping/Lying on side | Sleeping | 7 |
| Sleeping/Lying on side | Lying on my side | 3 |
| Sleeping/Lying on side | Lying on my side to sleep | 2 |
| Sleeping/Lying on side | Sleep comfortably | 6 |
| Sleeping/Lying on side | Sleeping on side (left) | 4 |
| Sleeping/Lying on side | Sleeping comfortably in bed | 5 |
| Sleeping/Lying on side | Sleeping on left or right side | 1 |
| squatting/lunging | Squats | 6 |
| squatting/lunging | Gym/involving squats | 3 |
| squatting/lunging | Gym/involving squats | 3 |
| squatting/lunging | Lunges at gym | 1 |
| squatting/lunging | Squatting | 4 |
| squatting/lunging | Performing weight bearing exercises | 2 |
| squatting/lunging | Squatting | 4 |
| squatting/lunging | Squatting | 2 |
| squatting/lunging | Squats and lunges | 4 |
| squatting/lunging | Lunges | 5 |
| squatting/lunging | Squatting, kneeling, rising | 4 |
| Stairs | Walking up steps | 7 |
| Stairs | Using the stairs | 5 |
| Stairs | Going up/down stairs | 6 |
| Stairs | Climbing stairs | 8 |
| Stairs | Carting groceries upstairs | 4 |
| Stairs | Going up stairs | 5 |
| Stairs | Stepping | 5 |
| Stairs | Up and down stairs | 2 |
| Stairs | Climbing stairs | 4 |
| Stairs | Steps at work | 5 |
| Stairs | Stairs at work with parcels | 7 |
| Stairs | Walking up stairs | 5 |
| Stairs | Walking up stairs | 3 |
| Stairs | Climbing flights of stairs | 5 |
| Stairs | Walking up stairs | 5 |
| Stairs | Walking up stairs | 2 |
| Stairs | Climbing stairs | 7 |
| Stairs | Stairs | 8 |
| Stairs | Climbing stairs | 8 |
| Stairs | Stairs | 3 |
| Standing | Standing | 4 |
| Standing | Standing (e.g. socialising) | 8 |
| Standing | Standing for long periods | 4 |
| Standing | Stand up for a long time | 5 |
| Standing | Standing | 4 |
| Standing | Stand for long periods of time | 5 |
| Standing | Stand for period of time | 5 |
| Standing | Standing | 5 |
| Standing | Stand for long period | 2 |
| Standing | Standing still for 30 minutes | 5 |
| Stretching | Hip opening exercises in Yoga and Pilates | 5 |
| Stretching | Stretching or taking up Pilates/yoga | 0 |
| Stretching | Stretching exercises | 4 |
| Stretching | Stretching exercises | 10 |
| Stretching | Stretching | 4 |
| Swimming | Swimming - getting out of pool | 5 |
| Swimming | Swimming | 2 |
| Swimming | Swimming | 6 |
| Swimming | Swimming | 6 |
| Swimming | Swimming | 6 |
| Swimming | Swimming | 5 |
| Swimming | Swimming | 8 |
| Swimming | Swimming | 10 |
| Swimming | Swimming | 8 |
| Swimming | Swimming | 6 |
| Swimming | Swimming | 10 |
| Swimming | Swimming | 8 |
| Swimming | Swimming laps | 6 |
| Walking | Bushwalking | 6 |
| Walking | Walking | 6 |
| Walking | Walking for leisure for 1 hour | 5 |
| Walking | Walking or jogging with dogs | 4 |
| Walking | Fast Walking | 1 |
| Walking | Walk dog | 5 |
| Walking | Walking | 8 |
| Walking | Bushwalking - 1/2 day - day | 8 |
| Walking | Going for walks | 0 |
| Walking | Walking the dogs | 7 |
| Walking | Participate in walking | 3 |
| Walking | Walking uphill | 7 |
| Walking | Walking | 5 |
| Walking | Power walking 30 mins | 9 |
| Walking | Vigorous walking | 5 |
| Walking | Walking | 6 |
| Walking | Walking | 3 |
| Walking | Brisk walking | 2 |
| Walking | Treadmill walking | 3 |
| Walking | Long walks | 5 |
| Walking | Walking | 7 |
| Walking | Walking without pain | 7 |
| Walking | Walking | 6 |
| Walking | Treadmill walking | 3 |
| Walking | Walking | 5 |
| Walking | Walking long distances | 8 |
| Walking | Taking the dog for a 30 minute walk | 7 |
| Walking | Walking quickly and painlessly | 5 |
| Walking | Walking 5-6 kms a day | 5 |
| Walking | Walking | 8 |
| Walking | Walking for an extended period (more than 30 mins0 | 5 |
| Walking | Walking up stairs | 7 |
| Walking | Walking | 6 |
| Walking | Brisk walk 40 - 50 mins | 6 |
| Walking | Walking | 6 |
| Walking | Regular walking exercise | 2 |
| Walking | Walking | 10 |
| Walking | Walking | 5 |
| Walking | Walking | 10 |
| Walking | Walking for exercise | 5 |
| Walking | Walking fast | 0 |
| Walking | Walking | 5 |
| Walking | Walking long distances | 6 |
| Walking | Walk everyday to improve fitness | 5 |
| Walking | Walking | 7 |
| Walking | Walking | 6 |
| Walking | Walking | 8 |
| Walking | Walking | 6 |
| Walking | Brisk Walk >20 mins 5 days a wk | 5 |
| Walking | Walking | 4 |
| Walking | Walking the dog | 5 |
| Walking | Hiking | 5 |
| Walking | Walking | 3 |
| Walking | Long brisk walks | 0 |
| Walking | Walking | 6 |
| Walking | Race walking | 0 |
| Walking | Walking up hill | 8 |
| Walking | Walking for more than 1 hour | 5 |
| Walking | Wearing high heels | 6 |
| Walking | Bushwalking | 7 |
| Walking | Walking | 5 |
| Walking | Walk any distance | 0 |
| Walking | Hiking - vigorous including hills | 3 |
| Walking | Walking for longer periods | 2 |
| Walking | Walking | 7 |
| Walking | Fast paced walking more than 60 mins | 4 |
| Walking | Long walks | 4 |
| Walking | Walking | 7 |
| Walking | Treadmill/walking | 5 |
| Walking | Walking for shopping for 2 hours | 4 |
| Walking | Long walks | 4 |
| Walking | Walking/hiking | 6 |
| Walking | Walking | 3 |
| Walking | Walking | 7 |
| Walking | Walking on uneven terrain/hills | 5 |
| Walking | Walking briskly for 30-45mins | 6 |
| Walking | Bushwalking | 7 |
| Walking | Walking after sitting 30 mins | 5 |
| Walking | Morning walk | 7 |
| Walking | Stiffness when walking | 3 |
| Walking | Walking at work - can become hard | 6 |
| Walking | Walking the dogs | 5 |
| Walking | Walking for very long periods | 2 |
| Walking | Walking /playing with the kids | 3 |
| Walking | Walking for long periods (>2 hrs) | 4 |
| Walking | Walking | 6 |
| Walking | Walking over 1 hour | 5 |
| Walking | Walking - stroll/shopping | 6 |
| Walking | Bushwalking | 3 |
| Walking | Walking for very long periods | 5 |
| Walking | Taking long walks | 5 |
| Walking | Walking | 8 |
| Walking | Walking | 9 |
| Walking | walking | 7 |
| Walking | Fast walking | 8 |
| Walking | Walking the dog | 5 |
| Walking | Walking for long periods | 4 |
| Walking | Walking slowly | 4 |
| Walking | Brisk walking | 2 |
| Walking | Walking up hills | 5 |
| Walking | Walking | 8 |
| Walking | Walking 5-6kms | 6 |
| Walking | Walk long distances | 7 |
| Walking | Walking up stairs | 8 |
| Walking | Walking or standing for extended periods | 5 |
| Walking | Walking | 4 |
| Walking | Walking | 10 |
| Walking | Short city walks | 5 |
| Walking | Walking | 5 |
| Walking | Walking | 5 |
| Walking | Walking long distance | 6 |
| Walking | Walking for exercise | 7 |
| Walking | Walking | 7 |
| Walking | Walking long distances >5km | 1 |
| Walking | Walking | 8 |
| Walking | Walking up a hill | 5 |
| Walking | Walking | 7 |
| Walking | Walking | 9 |
| Walking | Walking | 8 |
| Walking | Walking | 5 |
| Walking | Walking | 9 |
| Walking | Walk dog | 5 |
| Walking | Long walks | 5 |
| Walking | Fast walking | 4 |
| Walking | Walking | 7 |
| Walking | Walk uphill | 3 |
| Walking | Walking quickly | 7 |
| Walking | Walking for leisure | 5 |
| Walking | Walking long distances | 6 |
| Walking | Walk long distances with dogs | 3 |
| Walking | Walking long distance | 7 |
| Walking | Hiking | 4 |
| Walking | Bush walking | 1 |
| Walking | Walking dogs | 6 |
| Walking | Walking at a fast pace | 2 |
| Walking | Walking on level ground no hills | 5 |
| Walking | Walking up hills | 7 |
| Work | Work | 9 |
| Work | Able to work and concentrate | 4 |
| Work | Working for more than 30 mins | 5 |
| PSFS = Patient specific functional scale | | |
